# Baicalein Inhibits Metastatic Phenotypes in Nasopharyngeal Carcinoma Cells via a Focal Adhesion Protein Integrin β8

**DOI:** 10.3390/ph15010005

**Published:** 2021-12-21

**Authors:** Pichamon Kiatwuthinon, Thana Narkthong, Utapin Ngaokrajang, Supeecha Kumkate, Tavan Janvilisri

**Affiliations:** 1 Department of Biochemistry, Faculty of Science, Kasetsart University, Bangkok 10900, Thailand; fscipmk@ku.ac.th (P.K.); utapin.ng@ku.th (U.N.); 2 Department of Biochemistry, Faculty of Science, Mahidol University, Bangkok 10400, Thailand; thana_arm_91@hotmail.com; 3 Department of Biology, Faculty of Science, Mahidol University, Bangkok 10400, Thailand; supeecha.kum@mahidol.ac.th

**Keywords:** nasopharyngeal carcinoma, baicalein, metastasis, integrin β8, focal adhesion

## Abstract

Baicalein, a prominent flavonoid from the indigenous herbal plant *Scutellaria baicalensis* Georgi, possesses broad-spectrum anticancer activities. However, the biological effects of baicalein on nasopharyngeal carcinoma (NPC) and its underlying mechanisms remain unclarified. Thus, in this study, we examined the effects of baicalein on NPC cell lines and investigated the corresponding molecular mechanism through transcriptome profiling. In the study, four NPC cell lines were treated with various concentrations of baicalein at different time points. Cellular toxicity and proliferative inhibition of baicalein were examined by MTT assay. Metastatic phenotypes of NPC cells were investigated by wound healing, transwell, and adhesion assays. Additionally, microarray experiments were performed to determine the cellular pathways affected by baicalein. The expression and localization of the integrin β8 were validated by western immunoblotting and immunofluorescence. Our results revealed that baicalein exhibited its cytotoxicity and antiproliferative activity on all tested NPC cell lines. It also significantly inhibited metastatic phenotypes at sub-lethal concentrations. Transcriptomic analysis showed that baicalein significantly affected the focal adhesion pathway in NPC, where integrin β8 was greatly diminished. Thus, the present study results suggested that baicalein inhibits the metastatic phenotypes of NPC cells by modulating integrin β8, one of the major molecules in a focal adhesion pathway.

## 1. Introduction

Nasopharyngeal carcinoma (NPC) is a subtype of head and neck cancer arising from the mucosal epithelium of the nasopharynx. Although NPC incidence is rare in most parts of the world, it is highly prevalent in East and Southeast Asia [1,2]. The symptoms of NPC are not frequently shown and clearly identified owing to its latent development [2]. As a result, late diagnosis and low therapeutic efficacy are usually common, becoming the main obstacles for improving the treatment and enhancing survival rates in NPC patients. The etiology of NPC is complex and multifactorial, involving sex, environment, ethnicity, Epstein-Barr virus (EBV) infection, food consumption, and genome instability influences [2,3]. In many studies, NPC appears to be up to three-fold more prevalent in males than females [3]. Environmental factors including tobacco smoke, dust, and chemicals in the air play crucial roles in NPC carcinogenesis [1]. Individuals with East or Southeast Asian backgrounds exhibit a higher risk of NPC than other ethnic groups [2,4]. In addition, an association between NPC cases and EBV infection has been demonstrated. Mechanistically, EBV infection and reactivation trigger NPC pathogenesis [5,6]. Furthermore, foods with high content of 12-O-tetradecanoylphorbol-13-acetate (TPA), n-butyrate, nitrates, and nitrosamines, such as salted fish, meats, and vegetables, contribute greatly to the NPC development [6,7]. EBV reactivation, which could lead to genome instability and tumorigenesis, can be synergistically enhanced by a combination of chemicals such as TPA, sodium butyrate, and N-nitroso compounds [8]. Additionally, NPC tissues have revealed evidence of loss of heterozygosity at different chromosomal loci such as position 3p and 9p [9,10]. These multi-faceted risk factors distinctly affect the molecular characteristics of NPC cells, such as drug resistance, disease recurrence, and invasiveness, thus lowering the achievement of current treatment outcomes. Consequently, alternative measures are required to improve treatment efficiency. Natural and synthetically modified bioactive compounds have gained huge attention as their anticancer potentials have been demonstrated. They can be applied individually or in combination with current treatment methods to enhance the power of the cancer treatment synergistically [11,12,13,14].

Baicalein is one of the major bioactive flavonoids predominantly found in dried roots of the traditional herb *Scutellaria baicalensis*, Georgi [15]. It has various biological activities, including antiviral, antibacterial, anti-thrombotic, antidiabetic, anti-inflammation, and anticancer activities [16,17,18,19,20,21,22]. In addition, baicalein inhibits various types of cancers through distinct cellular signaling pathways [15,21,22,23,24,25,26,27]. Baicalein, for example, inhibits cell proliferation and induces apoptosis and senescence via the phosphatidylinositol 3-kinase/Akt signaling pathway in human colorectal cancer [21], via the Wnt/β-catenin pathway in osteosarcoma [27], via the caveolin-1/AKT/mTOR pathway in prostate cancer cells [24], and via the inhibition of Bcl-XL and Mcl-1 and activation of Bax, Bad, caspase-3, -8, and -9 in NPC [22]. Additionally, baicalein reduces metastatic potentials and epithelial-mesenchymal transition (EMT) of metastatic breast cancer cells [23]. For NPC, baicalein exerts its effect on anti-proliferation, cell cycle arrest, and apoptotic induction via the inhibition of EBNA1 Q-promoter activity [25]. However, the effects of baicalein on metastatic NPC phenotypes have not been investigated. Thus, in the present study, we investigated the effects of baicalein on NPC metastatic potentials, including cell migration, invasion, and adhesion, and elucidated the corresponding molecular pathways through genome-wide transcriptome analysis with the validation of a target protein candidate.

## 2. Results

### 2.1. Baicalein Exerts Its Cytotoxicity and Growth Inhibition on NPC Cell Lines

**Figure 1:**
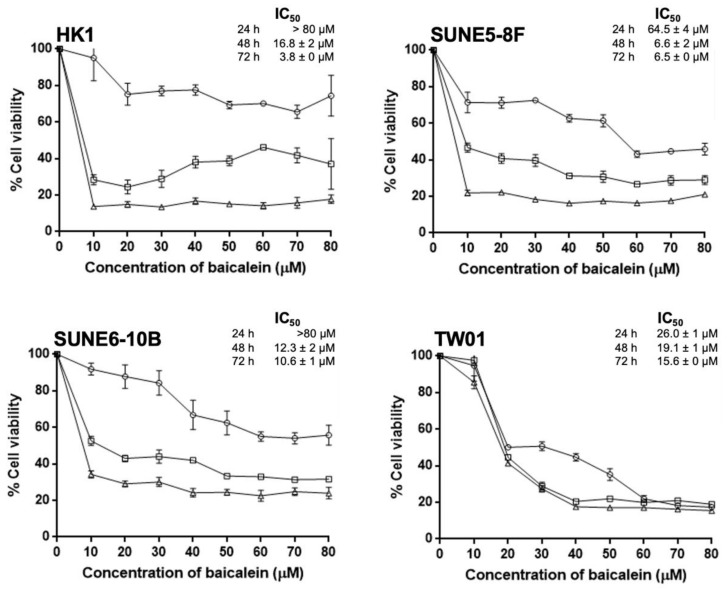
aGrowth inhibition profiles of four NPC cell lines, namely HK-1, SUNE 5-8F, SUNE 6-10B, and TW01, were determined by the MTT assay. All cells were treated with various concentrations of baicalein from 0 to 80 µM for 24, 48, and 72 h. The percentage of NPC cell viability was compared between baicalein-treated cells and the control. The IC_50_ values of baicalein inhibiting four NPC cell lines at 24, 48, and 72 h are shown. The IC_50_ values were obtained using the GraphPad Prism 6 program (San Diego, CA, USA). Data represent the mean ± standard error of the mean from three independent experiments.

Four NPC cell lines, namely HK-1, SUNE 5-8F, SUNE 6-10B, and TW01, were treated with various concentrations of baicalein ranging from 0 to 80 µM for 24, 48, and 72 h to evaluate the inhibitory effect of baicalein on NPC cell proliferation. The results exhibited that baicalein possessed growth inhibition activity in a dose- and time-dependent manner for all four NPC cell lines. Furthermore, an increase in baicalein incubation time resulted in significantly decreased IC_50_ values as observed in all NPC cell lines (Figure 1). In addition, HK-1 cells exhibited the highest time-dependent sensitivity to baicalein, while TW01 cells were slightly affected by the time course of baicalein treatment. These results indicate that baicalein exerts the growth inhibitory effects on NPC cells.

### 2.2. Baicalein Suppresses Cell Migration, Invasion, and Adhesion In Vitro

**Figure 2:**
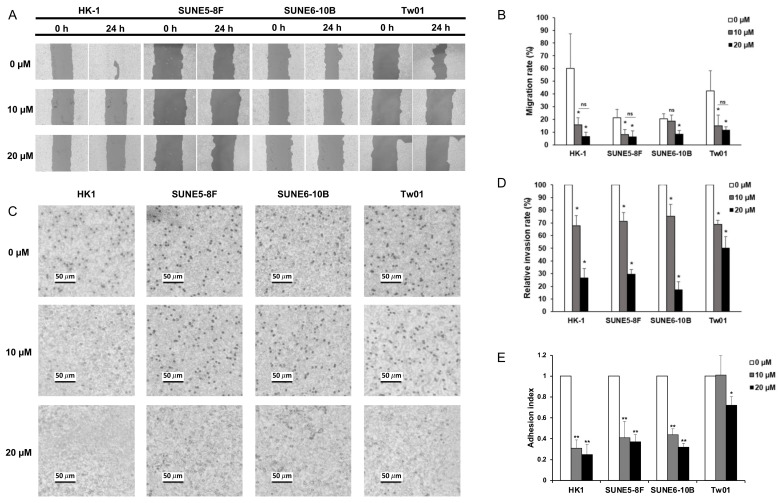
Baicalein inhibits NPC cell migration, invasion, and adhesion. (**A**) Wound-healing assay. NPC cells were seeded and cultured until confluence on the culture plate, and the wound was created by cell scratching for cell migration assay. Then, the cells were treated with 0, 10, and 20 µM of baicalein. The wound areas were observed at 0 h and 24 h with ×100 magnification using a light microscope, (Eclipse TS100; Nikon Corporation, Minato-ku, Tokyo, Japan), and (**B**) migration rates were represented. (**C**) In vitro invasion assay. The 8-well insert membrane was pre-coated by Matrigel before plating NPC cells. At 24 h, the invasive cells at the lower side of the insert membrane were fixed and stained with crystal violet. The images displayed the number of invasive cells at ×100 magnification using light microscope. (**D**) The invasive rate of NPC cells upon the exposure of different baicalein concentrations was determined by the ratio between the number of invasive cells in baicalein and those in the control groups. (**E**) Cell adhesion assay. NPC cells were resuspended with various concentrations of baicalein (0, 10, and 20 µM) and incubated for 6 h, then transferred to Matrigel-coated culture plate and cultured for 12 h. The cells re-adhering onto the ECM substrate were determined compared with the non-treated control cells. Data represent the mean ± standard error of the mean (SEM) from three independent experiments, ** *p* < 0.01 and * *p* < 0.05.

We further investigated the effect of baicalein on metastatic characteristics of NPC cells, including cell migration, invasion, and adhesion. For migration, NPC cells were pretreated with 10 and 20 µM baicalein, considered as sub-lethal doses of baicalein, for 24 h and were then wound-scratched by pipette tips. The areas of wound closure were determined at 24 h after treatment, and the relative areas of cell migration were calculated, compared to 0 h. The percentage of migration rate of all NPC cell lines was significantly reduced after exposure to baicalein, regardless of concentrations (Figure 2A,B). Interestingly, low metastatic SUNE 6-10B cells did not show a significant response on migration retardation at 10 µM, but the inhibition was substantially pronounced at 20 µM baicalein (*p* < 0.05). HK-1 cells, on the contrary, displayed a dramatic diminution in cell migration from 60.1% in control cells compared to 15.7% and 6.8% in 10 µM and 20 µM baicalein treatment, respectively. Moreover, baicalein dose-dependently suppressed NPC cell invasion (Figure 2C,D). The relative invasion rate revealed that all NPC cell lines responded to baicalein moderately at 10 µM and significantly at 20 µM of baicalein. Furthermore, cell adhesion, typically mediated through the binding of cell adhesion molecules on the cancer cell surface to the extracellular matrix at new distant organ sites, was evaluated at 24 h after the baicalein treatment [28]. After treatment with baicalein, NPC cells displayed a substantial reduction in adhesion index, suggesting that these baicalein-treated NPC cells lost their re-adhering ability onto the extracellular matrix (Figure 2E). HK-1 was the most sensitive cell line to baicalein, inhibiting cell adhesion. In contrast, TW01 cells exhibited the lowest sensitivity to baicalein, resulting in the re-adherence of cells onto the Matrigel substrate at 10 µM incubation. These functional investigations on the inhibition of cell migration, invasion, and adhesion revealed, for the first time, that baicalein also targets cell motility pathways in NPC. Consequently, HK-1 cells, the most subtle NPC cell line to baicalein, were selected as a model to explore further pathways attributed to baicalein-suppressing metastatic potentials using transcriptome analysis.

### 2.3. Transcriptome Analysis of NPC Cells after Baicalein Treatment Reveals the Suppression of Integrin β8 in a Focal Adhesion Pathway

**Figure 3:**
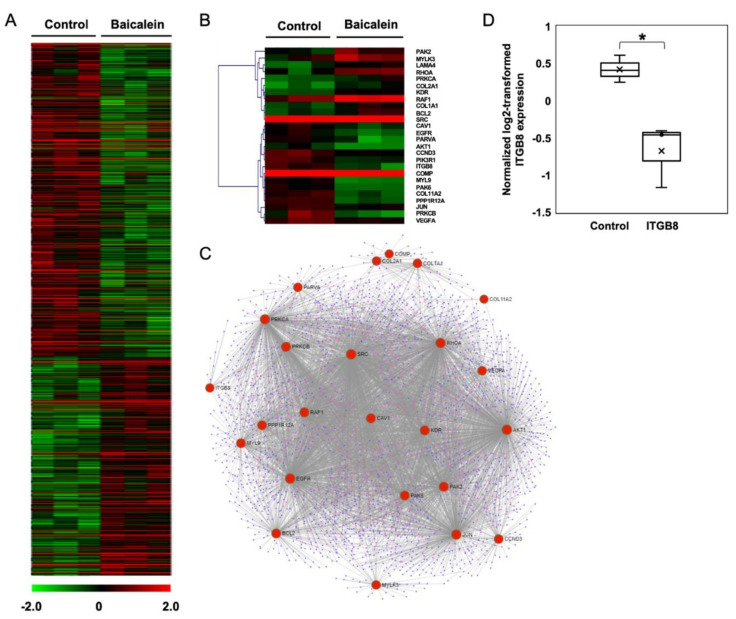
Transcriptomic profiles of NPC cells upon exposure to baicalein. (**A**) An illustration of a heatmap of hierarchical clustering of all 1274 DEGs between baicalein-treated and control HK-1 cells. (**B**) A heatmap of DEGs between the baicalein-treated and cell control groups, which are involved in the focal adhesion pathway as enriched by KEGG pathway analysis. The heatmaps were constructed using the hierarchical clustering function in the MeV program at a level of confidence * *p* < 0.05. As indicated in the diagram, a gradient of colors from red to green represents upregulation to downregulation. (**C**) An undirected protein-protein interaction network of DEGs with nodes highlighted on genes in the focal adhesion pathway using Network Analyst software. (**D**) A box plot of normalized log2-transformed values of integrin β8 was obtained from transcriptome analysis.

The transcriptome analysis of HK-1 cells was performed using whole-genome DNA microarrays. This high-throughput approach allowed us to uncover the alteration of genes and pathways in NPC cells in response to baicalein. The significant cluster analysis of baicalein-treated HK-1 cells revealed 1,274 DEGs compared to the control HK-1 cells (*p* < 0.05). There were 751 (58.9%) significantly upregulated, and 523 (41.1%) significantly downregulated DEGs, shown in a hierarchically clustered heatmap (Figure 3A). GO enrichment analysis demonstrated that two GO terms in a molecular function category were distinctively related to cell adhesion, including integrin binding and cadherin binding involved in cell-cell adhesion. Furthermore, KEGG pathway enrichment analysis represented that focal adhesion, PI3K-Akt, ErbB, TGF-β, and HIF-1 signaling pathways are major pathways that are evidently associated with cell migration, invasion, and adhesion (Table 1). 

A heatmap of the focal adhesion pathway represented 26 DEGs, comprising 11 upregulated and 15 downregulated genes (Figure 3B). The upregulated DEGs were *BCL2*, *COL1A1*, *COL2A2*, *KDR*, *LAMA4*, *MYLK3*, *PAK2*, *PRKCA*, *RAF1*, *RHOA*, and *SRC*. The downregulated DEGs included *ITGB8*, *COMP*, *PRKCB*, *PPP1R12A*, *MYL9*, *PARVA*, *COL11A2*, *PAK6*, *EGFR*, *AKT1*, *PIK3R1*, *CCND3*, *CAV1*, *VEGFA*, and *JUN*. Furthermore, a protein-protein interaction network (PPI) was used to explore the relationship between enriched DEGs in the focal adhesion pathway (Figure 3C). A total of 24 nodes were extracted from the enriched DEGs in the focal adhesion pathway. Among the protein interactions, we observed that integrin β8 (ITGB8) directly interacted with several downregulated proteins such as COMP, PRKCB, and PARVA. Based on the inhibition of cellular phenotypes, including cell migration, invasion, and adhesion, together with the bioinformatics data, we thus focused on integrin β8 protein, one of the essential cell adhesion molecules, which plays a vital role in regulating communication between cells and the ECM. According to the transcriptome profile of the baicalein-treated HK-1 cells, the normalized expression of integrin β8 was significantly lower than that of HK-1 control cells (Figure 3D). We further validated the expression and localization of integrin β8 in baicalein-treated HK-1 cells.

### 2.4. Integrin β8 Is Suppressed after Baicalein Treatment

**Figure 4:**
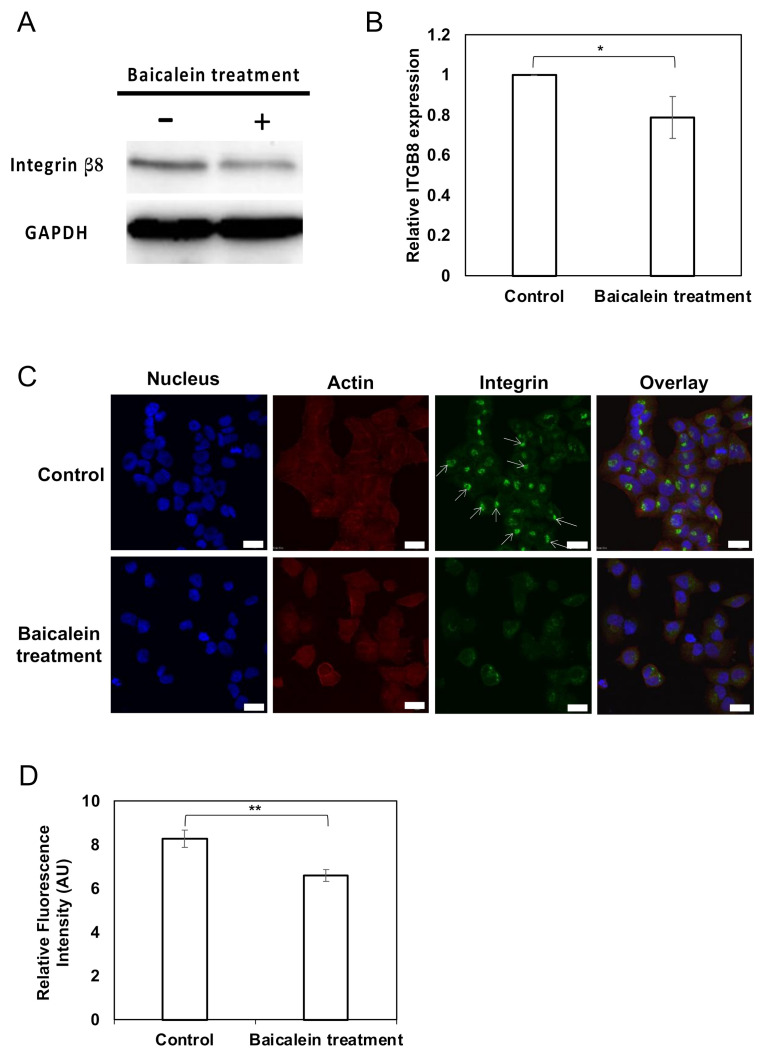
Validation of integrin β8 expression in baicalein-treated NPC cells. (**A**) Protein expression of integrin β8 in HK-1 cells with and without baicalein treatment. GAPDH was used as a loading control. Blots were cropped from the same gel to improve the clarity of protein-specific bands. (**B**) The relative protein expression of integrin β8 was determined by Western immunoblotting. (**C**) Immunofluorescence staining of intracellular integrin β8 protein in HK-1 cells. HK-1 cells were treated with 10 µM baicalein for 24 h and fixed with 4% paraformaldehyde. The cells were then stained with primary integrin β8 antibody, followed by Alexa-488-conjugated anti-mouse IgG. DAPI and TRITC-labeled phalloidin were used to counterstain the nucleus and actin, respectively. The arrows indicate the distinct pattern of integrin β8 in control HK-1 cells. Scale bar represents 20 µm. (**D**) The relative fluorescence intensity of baicalein-treated HK-1 cells compared to the control was determined by ImageJ software. All data represent the mean ± standard error of the mean (SEM) from three independent experiments, ** *p* < 0.01 and * *p* < 0.05.

We further validated and confirmed the downregulation of integrin β8 by Western immunoblotting. Indeed, the protein expression of integrin β8 was significantly reduced in baicalein-treated HK-1 cells (Figure 4A,B). We also determined the cellular localization of integrin β8 using an immunofluorescence staining technique (Figure 4C,D). The integrin β8 protein, actin filaments, and nucleus were visualized by Alexa-488-conjugated secondary antibody, TRITC-labeled phalloidin, and DAPI, respectively. In the control HK-1 cells, integrin β8 showed a dense and confined pattern of Alexa-488 fluorescence intensity close to the nucleus (Figure 4C, arrow pointed). On the contrary, baicalein-treated HK-1 cells had a distinct pattern of integrin β8 observed throughout the cytoplasm with faint fluorescence intensities. Moreover, the relative fluorescence intensity of integrin β8 in baicalein-treated HK-1 cells was significantly lower than that in the control HK-1 cells, ensuring the suppression of the integrin β8 production and redistribution of the protein after the baicalein treatment (Figure 4C,D).

## 3. Discussion

Current NPC treatment encounters low therapeutic efficacy in patients who are diagnosed at a later stage, causing a relapse with metastasis and lower survival outcomes [29]. Recently, naturally active compounds have been in the spotlight to alleviate these issues and reduce the side effects of current chemotherapeutic drugs and radiotherapy [30]. Baicalein, a flavonoid compound from dry roots of *S. baicalensis*, has anticancer activities both in vitro and in vivo. Baicalein exerted inhibitory effects on cell growth and cell cycle of numerous cancers through various molecular pathways such as PI3K/Akt [31], Wnt/β-catenin [27], and caveolin-1/AKT/mTOR [24], and the upregulation of p53 and caspases [22]. A previous study on NPC with baicalein showed that a decrease in cell viability of an EBV-positive NPC cell line was mediated through the downregulation of transcription factor specificity protein 1 (Sp1) and reduction in the EBNA1 activity in a dose- and time-dependent manner [25]. Meanwhile, an EBV-negative HK-2 cell line only demonstrated a minor growth-inhibiting effect. However, our findings with four EBV-negative NPC cell lines, including SUNE 5-8F, 6-10B, Tw01, and HK-1, showed that baicalein effectively exerts an antiproliferative effect. In fact, the reduced IC_50_ values confirmed the inhibition with a dose- and time-dependent manner. These results infer the potential of employing baicalein to treat both EBV-associated and EBV-non-associated NPC, which possess different chromosomal and molecular abnormalities [25].

For the first time, we demonstrated anti-metastasis activities of baicalein on NPC, including cell migration, invasion, and adhesion. Similar to our findings, baicalein displayed its anti-cell mobility activities in different types of cancer via various specific target molecules and pathways. Baicalein inhibited the migration of MDA-MB-231 breast cancer cells by suppressing SATB1, a nuclear matrix binding protein, and the Wnt/β-catenin pathway in a dose-dependent manner [32]. In addition, pancreatic cancer cell mobility was suppressed by baicalein through the downregulation of the PI3/Akt and MEK/ERK signaling pathways [33]. Moreover, the invasion of cancers such as breast, hepatoma, and ovarian cancer cells was markedly inhibited by baicalein by reducing matrix metalloproteinase (MMP)-2/-9 expression, associated with the MAPK signaling pathway [34,35,36]. Along with cell invasion and migration, baicalein exhibited its suppressive effect on the adhesion of cancer cells to the extracellular matrix, described as an early stage in establishing metastasis at secondary tumor sites [36,37]. Although controversial, the loss of cell-ECM adhesion has been demonstrated in vivo to mitigate cancer progression [27,38]. Furthermore, gene expression profiles of baicalein-treated non-small cell lung cancer cells unveiled the substantial downregulation of integrin alpha 1, 2, and 4 subunits, in agreement with anti-tumor growth in vivo [39].

According to our results, the GO annotation of DEGs also disclosed that “integrin binding” and “cadherin binding involved in cell-cell adhesion” were enriched in the molecular function category, associating with cell mobility. KEGG enrichment analysis further addressed that the focal adhesion and PI3K/Akt pathways were the two most significant signaling pathways targeted by baicalein. The enriched PI3K/Akt pathway has also been consistently reported as a typically targeted pathway for baicalein in many studies, including pancreatic [33], esophageal squamous [31], melanoma [40], breast [36], and colorectal cancers [41]. Additionally, both focal adhesion and PI3K/Akt pathways share some key central proteins, indicating a strong correlation between these two pathways for inhibiting cancer mobility [24,40]. We focused on integrin β8 protein, one of the focal adhesion molecules bridging the communication between cells and the extracellular matrix, downregulated at both gene and protein levels. Furthermore, integrin β8 interconnects with several proteins, facilitating the control of focal adhesion. Our PPI analysis disclosed the interconnection between ITGB8 and several proteins, such as COMP, involving the production of cartilage oligomeric matrix protein. Similar to the present data, a meta-analysis in lung adenocarcinoma patients also exhibited a strong relationship between ITGB8 and COMP, contributing to the regulation of lung cancer metastasis and poor treatment outcomes [42]. Moreover, an integrin β8 was involved in regulating migration and invasion in several types of cancer including glioblastoma, prostate, lung, and ovarian cancers [43,44,45,46]. Integrin β8-overexpressing metastatic glioblastoma cells reinforced the invasion by forming a complex between integrin β8 and Rho-GDP dissociation inhibitor 1 (RhoGDI1) [44]. In contrast, a knockdown of integrin β8 or an interruption between integrin β8 and RhoGDI1 could reverse the cancer cell invasion via prohibiting Rho GTPase and increasing the GTP-bound Rho proteins [44]. Another study in cisplatin-resistant ovarian SKOV3 cancer cells uncovered that the suppression of integrin β8 by miR-199a-3p diminished cancer cell invasion [47]. In fact, integrin β8 displays diverse functions, in addition to the inhibition of cell mobility, including the enhancement of cell cycle arrest, apoptosis, and drug sensitization [47,48]. In our study, the distinct subcellular localization of integrin β8 where the perinuclear accumulation vanished after the baicalein treatment was observed. The translocation of integrin β8 from perinuclear to cytosol regions was shown to colocalize with the Golgi apparatus in pancreatic cancer cells after exposure to gemcitabine and X-ray [48]. In the same study, the International Cancer Genome Consortium (ICGC) and COSMIC database were explored to confirm that no gene alteration of integrin β8 in pancreatic cells that could cause abnormal localization patterns. Additionally, the distribution of integrin β8 in the cytoplasm was presented in normal microvascular endothelial cells, suggesting cytoplasmic subcellular localization of integrin β8 [49]. Nevertheless, a profound mechanistic study of the reorganization pattern of intracellular integrin β8 in NPC related to baicalein exposure requires further investigation.

Despite being a potent anticancer agent, baicalein at micromolar ranges is typically employed in in vitro studies [50]. This is considered as a limitation in translating the use of baicalein in vitro to in vivo and clinical practice, where the nanomolar range is usually observed [51,52]. Pharmacokinetic studies of baicalein have been extensively performed in rodents, monkeys, and humans, which revealed drug excretion through urine and feces and chemical modification of baicalein by glucuronidation [51,52,53,54,55]. A pharmacokinetic study in humans orally consuming up to 2800 mg baicalein exhibited *T*_max,_ *t*_1/2_, and *C*_max_ of 0.75 h, 15.01 h, and 108.17 ng/mL, respectively, indicating a reduction in baicalein in blood plasma. Remarkably, the baicalein administered was tolerated and safe for human health [54]. Consequently, further optimization on drug efficacy and stability, and safety is essential for clinical translation [56]. Baicalein can be chemically modified to obtain derivatives with improved pharmacokinetics and pharmaceutical properties, including anti-proliferation [12]. Alternatively, the efficacy and permeability of baicalein in vivo can be enhanced using engineered delivery platforms such as baicalein-encapsulating lipid and polymer nanoparticles [57,58]. The lipid nanoparticles increase the bioavailability of entrapped baicalein by more than 300% [57]. Thus, it is plausible to exploit baicalein as an anti-metastatic agent in vivo for treating metastatic NPC; however, further investigations on this compound are warranted. Altogether, we demonstrate that baicalein downregulates integrin β8 in NPC cells and suppresses NPC metastatic phenotypes, thereby potentially improving patient survival rates and NPC therapeutic efficiency.

## 4. Materials and Methods

### 4.1. Chemicals and Cell Line Maintenance

All chemicals, unless indicated otherwise, were purchased from Sigma-Aldrich (St. Louis, MO, USA). Baicalein (98% purity) (Product No.: 465119) was prepared in dimethyl sulfoxide (DMSO) at 100 mM as stock solution and stored at −20 °C. Desired concentrations of baicalein were freshly prepared by diluting the stock solution in a serum-free medium, DMSO 1% (*v*/*v*) was used as a control throughout all experiments. NPC cell lines in this study included HK-1 (kindly provided by Prof. Maria L Lung, University of Hong Kong), SUNE 5-8F, SUNE 6-10B (kindly obtained from Prof. Qingling Zhang, Southern Medical University), and TW01 (kindly gifted from Prof. C-T Lin, National Taiwan University). HK-1, SUNE 5-8F, and SUNE 6-10B were cultured in Roswell Park Memorial Institute medium (RPMI-1640; Invitrogen, NY, USA) with 10% fetal bovine serum (FBS; Thermo Scientific Hyclone, UT, USA) and 100 U/mL penicillin, 100 µg/mL streptomycin (Invitrogen, NY, USA), incubated at 37 °C with 5% CO_2_. Tw01 cells were cultured in Dulbecco’s modified Eagle medium supplemented with 10% FBS and 100 U/mL of penicillin, 100 µg/mL of streptomycin at 37 °C with 5% CO_2_. All cell lines were sub-cultured at 80–90% confluence, and the media were replaced every 48 h.

### 4.2. MTT Assay

Approximately 3 × 10^3^ cells/well were seeded in a 96-well plate (Corning, NY, USA) and cultured for 24 h. Cells were then washed, incubated with various concentrations of baicalein (0 to 80 µM) for 24, 48, and 72 h. After that, the cells were incubated with 0.5 mg/mL 3-(4, 5-dimethylthiazol-2-yl)-2, 5-diphenyltetrazolium bromide (MTT) (United States Biological, MA, USA) for 3 h. The medium was then replaced with DMSO, and a microplate reader measured the absorbance at 540 nm (PerkinElmer’s VICTOR4; Perkinelmer, Inc., Waltham, MA, USA). The percentage of cell viability was calculated as the ratio of absorbance of treated cells to the absorbance of the untreated cells. The half-maximum inhibitory concentration (IC_50_) values were calculated by fitting the data into a nonlinear regression using GraphPad Prism 6 software. All experiments were independently performed in triplicate.

### 4.3. Cell Migration by Wound-Healing Assay

A total of 2 × 10^5^ cells/well were seeded into a 24-well plate and cultured until confluent. Wounds were scratched using 200 µL sterile pipette tips. The cells were then incubated with 10 and 20 µM baicalein, which was sub-lethal baicalein concentrations for the cells, in 0.1% FBS-containing media for 24 h. Images were obtained by a phase-contrast inverted microscope (Eclipse TS100; Nikon Corporation, Minato-ku, Tokyo, Japan) at 0 and 24 h. The cell migration rates were examined from the measurement of the wound area of experimental groups from *t* = 0 and *t* = 24 h compared to that of the untreated control group using TScratch software. Three independent experiments were performed in triplicate, and the data are expressed as the mean ± SEM.

### 4.4. Cell Invasion Assay

Approximately 1 × 10^5^ cells/well were seeded into a 6-well plate and then cultured for 24 h. Next, the cells were harvested and adjusted to a density of 1 × 10^4^ cells/ 100 µL before being exposed to 10 and 20 µM baicalein in serum-free medium for 6 h. Following the incubation, cell suspensions were added onto an 8 µM-pore PET membrane chamber (EMD Millipore, MA, USA), pre-coated with Matrigel (Corning, NY, USA). A medium supplemented with 10% FBS as a chemoattractant was added to the lower PET insert chamber. The non-invaded cells on the upper surface were scraped off by the cotton swab after 12 h. Invading cells located on the lower surface of the membrane were fixed with 4% paraformaldehyde for 20 min and stained with 0.5% crystal violet solution overnight. The stained cells were photographed and counted under an inverted light microscope. The relative cell invasion rate was determined as the number of invaded cells in baicalein treatment groups compared to that in a non-treated control group. Three independent experiments were performed in triplicate, and the data are expressed as the mean ± SEM.

### 4.5. Cell Adhesion Assay

Matrigel was pre-coated and air-dried in a 96-well plate. NPC cells were harvested, and 1 × 10^4^ cells were resuspended in different concentrations of baicalein-containing media and then further incubated for 6 h. After that, cells were plated onto a pre-coated Matrigel^TM^ surface and further incubated for 12 h. The unadhered cells were removed by washing with PBS three times, and the re-adhered cells onto the matrigel-coated plate were determined by the MTT assay as described previously. Cell adhesion index was obtained from the ratio between the number of re-adhering cells in experimental groups to that in a control group. Three independent experiments were performed in triplicate, and the data are expressed as the mean ± SEM.

### 4.6. Protein Extraction and Western Blotting

NPC cells (1 × 10^6^ cells/10 mL medium) were plated onto a 10 cm Petri dish and cultured for 24 h before exposure to 10 µM baicalein for 24 h. They were washed with PBS and lysed with ice-cold RIPA lysis buffer (50 mM Tris-HCl, 150 mM NaCl, 5 mM EDTA, 0.1% SDS, 0.5% sodium deoxycholate and 1% NP-40 with 1 mM PMSF). To remove cell debris, the samples were centrifuged at 9000× *g* for 20 min at 4 °C. The protein concentrations were determined by Bradford assay (Bio-Rad, CA, USA). Total proteins were then separated by SDS-PAGE and transferred onto nitrocellulose membranes. Nonspecific binding was blocked with 10% BSA for 2 h at room temperature. The primary antibodies against ITGB8 (ab80673; Abcam, MA, USA) (1:1000), β-actin (A1978; Sigma-Aldrich, MO, USA) or and GAPDH (ab128915; Abcam, MA, USA) (1:20,000) were incubated with the membrane overnight at 4 °C. The secondary anti-rabbit antibody conjugated with horseradish peroxidase (HRP) (1:1000) (Cell Signaling Technologies, MA, USA) was added and further incubated for 30 min. The immunoreactivities were detected by SignalFire™ ECL Reagent (Cell Signaling Technologies). The experiments were independently performed in triplicate. The intensities of bands were evaluated using the ImageJ program (Version 1.52r) [59].

### 4.7. Microarray Hybridization

NPC cells were incubated with 10 µM baicalein for 24 h. Total RNA was extracted using E.Z.N.A.^®^ Total RNA Kit I (Omega Biotek, Norcross, GA, USA) according to the manufacturer’s instructions. Total RNA samples were then treated with DNAse I (Omega Biotek), purified, and spectrophotometrically quantified at 260 nm using NanoDrop™ UV-Vis Spectrophotometer (Thermo Scientific™, Waltham, MA, USA). RNA samples with an absorbance ratio of 260/280 nm higher than 1.8 were subjected to a labeling process described previously [60]. Briefly, 6 to 10 µg of total RNA was reverse-transcribed by reverse transcriptase (Thermo Scientific™) in the presence of 7 µg of Cy3-labeled random nonamers (Integrated DNA Technologies, Singapore), 3.33 mM dNTP, Ribolock nuclease inhibitor, and 5 mM DTT (Thermo Scientific™). The reaction mixture was incubated at 25 °C and 42 °C for 5 and 180 min, respectively. Then, 200 mM NaOH and 20 mM EDTA were added at a 1:1 ratio to remove the RNA template strands. The reaction was incubated at 65 °C for 10 min; then, one volume of 1 M HEPES buffer was added. The Cy3-labeled cDNA strands were purified by a GeneJET DNA purification kit (Thermo Scientific™). The OneArray^®^ Human Microarray v5 (HOA 5.1) (PhalanxBio, Inc., CA, USA) were pre-warmed and pre-hybridized at 60 °C for 10 min and 42 °C for 1 h, respectively. The arrays were then rinsed with ultrapure water and spun to eliminate water residue. Next, they were incubated and hybridized with Cy3-labeled cDNA strands in a hybridization chamber saturated with 2× SSC at 42 °C for 16–18 h. After incubation, the arrays were washed and rinsed with 1× SSC buffer twice, followed by 0.1× SSC buffer. Then, the intensity of Cy3-hybridized arrays was scanned by a microchip LS Reloaded scanner (Tecan Group Ltd., Switzerland). A 532 nm laser was used for the scan with 6 µm resolution and 220 PMT gain. The intensities from the scanner were processed and quantified with Array Pro Analyzer software. Microarray data were deposited in the Gene Expression Omnibus with the accession number GSE127765.

### 4.8. Data Processing and Analysis

The microarray data from three biological replicates were obtained as raw intensity format, transformed by log2 value, median, and quantile normalization, respectively. Differentially expressed genes (DEGs) were obtained from an equal variance t-test between control and 10 µM baicalein-treated groups. The biological significance of DEGs was then explored using DAVID bioinformatics resources version 6.8 [61]. Gene ontology (GO) term and the Kyoto Encyclopedia of Genes and Genomes (KEGG) pathway enrichment analyses were performed. DEGs were clustered based on their molecular functions annotated by GO terms. Pathway enrichment analysis was used to identify important pathways that were altered as a result of baicalein treatment. A confidence level at *p* < 0.05 was employed to obtain significant DEGs and enrichment analysis. For protein interaction network analysis, the DEGs from the focal adhesion pathway were used for the construction of the protein-protein interaction (PPI) networks using Network Analyst [62].

### 4.9. Immunofluorescence

NPC cells (1 × 10^5^ cells/well) were plated on 8-well chamber slides (SPL Life Science, South Korea) for 24 h. The cells were treated with 10 µM baicalein for 24 h. Cells in 1% DMSO were used as a control. They were washed, fixed with 4% paraformaldehyde, permeabilized with 0.1% Triton X-100, blocked with 10% FBS, and probed with 4.5 µg/mL rabbit polyclonal anti-integrin β8 antibody overnight (ab80673; Abcam, Cambridge, MA, USA) (1:100). The cells were incubated with 5 µg/mL Alexa-488-conjugated goat anti-rabbit IgG secondary antibody (A-11034; Thermo Scientific™) (1:100) for 1 h. The cells were then counterstained with 1 µg/mL Tetramethylrhodamine (TRITC)-conjugated phalloidin and 5 µg/mL DAPI (AppliChem, Germany) before imaging using an FV1000 confocal microscope (Olympus, USA).

### 4.10. Statistical Analysis

Results were expressed as mean ± stand error of the mean (SEM) of triplicate independent experiments. A two-tailed unpaired t-test was used to compare the control and sample groups. A confidence level of >95% indicated statistical significance for all experiments (** *p* < 0.01; * *p* < 0.05; “ns” indicates not significant).

## 5. Conclusions

For the first time, our present work reported the inhibitory effect of baicalein on NPC cell migration, invasion, and adhesion. Transcriptome analysis revealed that baicalein exerted anti-metastasis activities in NPC through the suppression of focal adhesion and PI3K/Akt pathways. In addition, we unveiled that the downregulation of integrin β8, one of the key cell adhesion proteins in the focal adhesion and PI3K/Akt pathways, as evidenced in both gene and protein expression levels. Thus, we propose integrin β8 as a potential targeted protein in NPC in response to baicalein exposure, and it can be used as a therapeutic marker in the forthcoming NPC treatment.

## Figures and Tables

**Table 1:** Selective KEGG pathway enrichment analysis of all differentially expressed genes of baicalein-treated NPC.

KEGG Pathways	Gene Counts	*p* Values	Gene Names
Focal adhesion	26	2.33 × 10^−4^	*CAV1*, *COL2A1*, *SRC*, *MYL9*, *PAK6*, *AKT1*, *PAK2*, *ITGB8*, *COMP*, *BCL2*, *RHOA*, *PPP1R12A*, *COL11A2*, *PIK3R1*, *EGFR*, *PRKCA*, *MYLK3*, *RAF1*, *KDR*, *PRKCB*, *LAMA4*, *CCND3*, *JUN*, *VEGFA*, *COL1A1*, *PARVA*
PI3K-Akt signaling pathway	37	2.43 × 10^−4^	*FGFR2*, *PPP2R3A*, *FGF9*, *FGF10*, *COL2A1*, *GNG8*, *AKT1*, *ITGB8*, *BCL2*, *COMP*, *PPP2CB*, *CSF3R*, *COL11A2*, *FGF1*, *PIK3R1*, *SYK*, *GNG7*, *PRKCA*, *EGFR*, *PPP2R1A*, *IL2RA*, *CREB1*, *YWHAB*, *PKN2*, *RAF1*, *BCL2L11*, *KDR*, *NRAS*, *GNGT1*, *LAMA4*, *CDKN1A*, *CCND3*, *CHRM2*, *VEGFA*, *JAK1*, *MDM2*, *COL1A1*
Oxytocin signaling pathway	20	7.67 × 10^−4^	*PRKCA*, *EGFR*, *ADCY1*, *MYLK3*, *CACNG7*, *PRKAG2*, *RAF1*, *OXTR*, *KCNJ3*, *SRC*, *PRKCB*, *MYL9*, *NRAS*, *CDKN1A*, *JUN*, *RHOA*, *PPP1R12A*, *CAMK2D*, *PPP3CC*, *RYR2*
Renal cell carcinoma	12	1.08 × 10^−3^	*PAK6*, *AKT1*, *NRAS*, *CUL2*, *HIF1A*, *PAK2*, *EPAS1*, *VHL*, *JUN*, *VEGFA*, *RAF1*, *PIK3R1*
ErbB signaling pathway	14	1.13× 10^−3^	*PRKCA*, *EGFR*, *RAF1*, *SRC*, *PRKCB*, *AKT1*, *PAK6*, *NRAS*, *CDKN1A*, *EREG*, *PAK2*, *JUN*, *CAMK2D*, *PIK3R1*
Pathways in cancer	38	1.43 × 10^−3^	*FGFR2*, *ADCY1*, *FGF9*, *FGF10*, *CXCL12*, *GNG8*, *AKT1*, *CUL2*, *CDKN2B*, *BCL2*, *RHOA*, *CSF3R*, *RARB*, *RUNX1*, *FGF1*, *PIK3R1*, *GNG7*, *PRKCA*, *EGFR*, *PTGER1*, *BMP2*, *PTGER4*, *EPAS1*, *VHL*, *RAF1*, *FZD5*, *CTNNA3*, *PRKCB*, *DAPK1*, *NRAS*, *GNGT1*, *LAMA4*, *CDKN1A*, *HIF1A*, *JUN*, *VEGFA*, *JAK1*, *MDM2*
TGF-β signaling pathway	13	2.57 × 10^−3^	*PPP2R1A*, *BMP2*, *SMAD5*, *DCN*, *ACVR2A*, *SP1*, *CDKN2B*, *ZFYVE16*, *PPP2CB*, *RHOA*, *TGIF1*, *ACVR1*, *BMP8A*
HIF-1 signaling pathway	14	2.80 × 10^−3^	*PRKCA*, *EGFR*, *VHL*, *PFKFB3*, *PRKCB*, *AKT1*, *CUL2*, *CDKN1A*, *HIF1A*, *BCL2*, *VEGFA*, *CAMK2D*, *PIK3R1*, *NPPA*
Dopaminergic synapse	16	5.66 × 10^−3^	*PRKCA*, *PPP2R1A*, *PPP2R3A*, *CALY*, *CREB1*, *TH*, *GRIA3*, *KCNJ3*, *PRKCB*, *AKT1*, *GNG8*, *GNGT1*, *PPP2CB*, *CAMK2D*, *PPP3CC*, *GNG7*
Viral carcinogenesis	22	5.91 × 10^−3^	*CREB1*, *YWHAB*, *SNW1*, *SRC*, *NRAS*, *CDKN1A*, *CCND3*, *CDKN2B*, *GSN*, *JUN*, *RHOA*, *JAK1*, *MDM2*, *HIST1H4C*, *HIST1H4I*, *ATP6V0D1*, *HDAC9*, *ATP6V0D2*, *HDAC8*, *PIK3R1*, *SYK*, *HIST1H4H*

## Data Availability

All data sets during and/or analyzed during the current study are available from the corresponding author upon request. All microarray data were deposited in the Gene Expression Omnibus with the accession number GSE127765.

## Abbreviations

COSMIC	Catalog of somatic mutations in cancer
DAPI	4′,6-Diamidino-2-Phenylindole
DEGs	Differentially expressed genes
DMSO	Dimethyl sulfoxide
EMT	Epithelial-mesenchymal transition
EBV	Epstein-Barr virus
FBS	Fetal bovine serum
GO	Gene ontology
ICGC	International Cancer Genome Consortium
KEGG	Kyoto Encyclopedia of Genes and Genomes
NPC	Nasopharyngeal carcinoma
PPI	Protein-protein interaction
SEM	Stand error of mean
TRITC	Tetramethylrhodamine-isothiocyanate
TPA	12-*O*-tetradecanoylphorbol-13-acetate
